# Potato improvement through genetic engineering

**DOI:** 10.1080/21645698.2021.1993688

**Published:** 2022-01-06

**Authors:** María del Mar Martínez-Prada, Shaun J Curtin, Juan J Gutiérrez-González

**Affiliations:** a Departamento De Biología Molecular, Facultad De Ciencias Biológicas Y Ambientales, Universidad De León, León, España; b United States Department of Agriculture, Plant Science Research Unit, Minnesota, USA; c Department of Agronomy and Plant Genetics, University of Minnesota, Minnesota, USA; d Center for Plant Precision Genomics, University of Minnesota, Minneapolis, Minnesota, USA; e Center for Genome Engineering, University of Minnesota, Minneapolis, Minnesota, USA

**Keywords:** Acrylamide, CRISPR/Cas9, Colorado potato beetle, gene editing, *Phytophthora infestans*, potato

## Abstract

Potato (*Solanum tuberosum* L.) is the third most important crop worldwide and a staple food for many people worldwide. Genetically, it poses many challenges for traditional breeding due to its autotetraploid nature and its tendency toward inbreeding depression. Breeding programs have focused on productivity, nutritional quality, and disease resistance. Some of these traits exist in wild potato relatives but their introgression into elite cultivars can take many years and, for traits such as pest resistance, their effect is often short-lasting. These problems can be addressed by genetic modification (GM) or gene editing (GE) and open a wide horizon for potato crop improvement. Current genetically modified and gene edited varieties include those with Colorado potato beetle and late blight resistance, reduction in acrylamide, and modified starch content. RNAi hairpin technology can be used to silence the haplo-alleles of multiple genes simultaneously, whereas optimization of newer gene editing technologies such as base and prime editing will facilitate the routine generation of advanced edits across the genome. These technologies will likely gain further relevance as increased target specificity and decreased off-target effects are demonstrated. In this Review, we discuss recent work related to these technologies in potato improvement.

## Introduction

Potato (*Solanum tuberosum* L.) is the third most important food for human consumption behind wheat and rice, and among the top horticultural crops.^1^ Two thirds of the annual yield is marketed fresh, while the remainder is processed for snack and other industrial food products, including animal feed, adhesives, pharmaceuticals, wood, and textile commodities.^2,3^ In 2019, 17.5 million hectares of potatoes were cultivated worldwide, yielding 370.5 million tons (Table 1).^4^ Asia is the world’s largest potato producer with more than 9 million hectares grown and 189 million tons harvested. Europe produced almost a third of the world total harvest (107 million tons) that was grown on more than 4.5 million hectares. Africa and North America have a similar hectarage dedicated to potato cultivation, however North American production is almost double that of Africa.^4^

**Table 1.** Area harvested in hectare and production in tons for each continent and world’s total. Data are about year 2019, which has been obtained from Food and Agriculture Organization Database

Regions	Area harvested (ha)	Production (t)
Africa	1,763,848	26,534,489
America (North & South)	1,539,393	45,083,546
Asia	9,298,106	189,810,377
Europe	4,696,336	107,264,935
Oceania	43,303	1,743,234
TOTAL	17,340,986	370,436,581

Potato was first domesticated in South America, in the Andes Mountain range between the border of Peru and Bolivia.^5^ However, its genomic characteristics and large population of wild species alongside modern cultivars have made the precise determination of its origin difficult.^6^ Potatoes were introduced into Spain during the second half of the 16th century, and spread to the rest of Europe shortly afterward.^7^

The Potato belongs to the Solanaceae family and are classified in the *Solanum* genus which is comprised of approximately 900 species. The genus can be further divided into the *Petota* clade, that includes all tuber producing species. Cultivated potato belongs to the species *Solanum tuberosum* first described by Carl von Linneo in 1753 and can be further classified into two subspecies. The first includes the cultivated varieties *S. tuberosum* subsp. *tuberosum*,^8^ whose life cycle is adapted to long days and likely originated from the first introduction into Europe. The second subspecies, *S. tuberosum* subsp. *andigena*, is comprised of South American landraces.^8^ Taxonomy of *Solanum* has been extensively studied and is still highly controversial. Despite the advances in taxonomy brought about by the development of molecular analysis tools, such as SNPs, AFLPs, RAPDs and next-generation sequencing, contradictions among researcher are frequent.^9^ To a large extent, this problem derives from the large number of local genetically diverse varieties present in Argentina, Bolivia, Mexico, and Peru, in addition to historical hybridization events.^8^

Cultivated potato is a highly heterozygous autotetraploid (2 n = 4x = 48) with twelve chromosomes. Its genome likely resulted from hybridizations between diploid wild potatoes undergoing chromosome duplication.^8^ Maintaining heterozygosity is essential to avoid problems associated with inbreeding depression, including reduced fertility and productivity.^10,11^ In agricultural production, the potato is grown as an annual crop with vegetative reproduction using tubers as planting materials, while reproduction by seed is limited to breeding programs.^12,13^

Potatoes are susceptible to a wide variety of biotic stresses with defoliating insects one of the major threats, reducing both productivity and quality of the tubers. Among the insect pathogens, the Colorado potato beetle (*Leptinotarsa decemlineata* Say) stands out due to its devastation and resistance to insecticides. Other insects feed on tubers, weakening the plant and leading to production losses and in some cases plant death.^14^ Furthermore, some insects can be problematic for their viral pathogen vector roles, notably the aphid-borne potato leaf roll virus (PLRV) and potato virus Y (PVY) that can cause yield losses of up to 80%.^15^ Potatoes are also affected by nematodes, and by several bacterial species such as *Ralstonia solanacearum*.^16–18^ Nevertheless, the pathogen that generates the most striking losses worldwide is the oomycete pathogen *Phytophthora infestans*, which causes late blight disease leading to premature plant death.^17,19,20^ Additional fungal pathogens of concern include *Alternaria solani*, numerous species of *Fusarium* and *Rhizoctonia*; and *Passalora concors*, which causes a disease called Cercospora leaf blotch.^17,20^ To protect the plant against these pests and pathogens requires numerous insecticidal and antifungal treatments throughout the growing season.

The increased susceptibility of potato to pest and disease is likely due to the loss of genetic diversity resulting from traditional breeding techniques. For example, selection of tubers with reduced bitterness has led to lower levels of the insect deterring glycoalkaloid metabolites in the leaves, thereby potentially exacerbating insect damage.^21,22^ Fortunately, because of the large number of potato species and landraces, there is a wide range of germplasm available to improve varietal characteristics, in particular resistance to pathogens.^23^ Related wild species provide a broad compatible gene pool ready to be used in breeding programs. However, despite the fact that 40% of non-domesticated species have traits of interests, their introgression into commercial varieties through traditional breeding is difficult. Many wild species have different ploidy levels than cultivated potato leading to sexual incompatibility and large phenotypic variation and unwanted effects.^2^ Furthermore, it is difficult to make individuals homozygous for all four copies of the gene of interest.^23^ Therefore, the application of genetic engineering technologies to potato could be a great benefit for its improvement.^2^

This review aims to highlight the current genetic engineering tools that are being employed in potato improvement, with special emphasis on varieties that have reached the market. It examines the traits that have been modified in potato, the methods used, and the final outcomes. Finally, future perspectives on the most promising gene editing techniques will be discussed.

## Methods of Genetic Modification in Plants

A genetically modified organism (GMO) can be defined as one whose genetic material has been altered using genetic engineering techniques. In the same way that the application of classical genetics in plant breeding was one of the main factors that led to the green revolution, biotechnology and genetic engineering has triggered a second green revolution. Genetic engineering originated in 1983 when the first exogenous DNA was introduced into a plant by transformation with *Agrobacterium tumefaciens*.^24,25^ This strategy consisted of modifying a bacterial (*Ti*) plasmid required to infect the plant by removing detrimental oncogene-related sequences and replacing them with gene-of-interest (GOI) sequences. *Agrobacterium*-mediated gene delivery was the first genetic modification system used in crops, starting a revolution which has altered the traditional landscape long established by plant breeding.^2^ There are two types of gene introductions; *trans*-genesis and *cis*-genesis. *Trans*-genesis involves introducing a gene from a sexually incompatible species, such as bacteria while *cis*-genesis uses DNA from the same or a closely related species, thereby reproducing a modification that could have occurred naturally.^26,27^

RNA interference (RNAi) technology in plants emerged some years later with the demonstration that gene silencing was initiated by double-stranded RNA (dsRNA).^28^ These initial experiments used sense and antisense sequences to degrade Potato Virus Y (PVY) viral RNA. This break-through would lead to the development of more efficient inverted repeat (IR) or hairpin RNA (hpRNA) transgenes that are used extensively for targeted silencing of genes in plants and animals today.^28–31^

**Figure 1.**
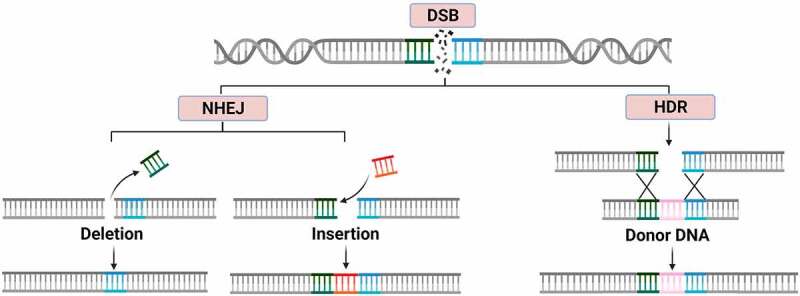
The repair pathways for double-stranded cuts in DNA. A) non-homologous end joining repair (NHEJ) that can produce insertions and deletions. B) an example of homology-directed repair (HDR) inserting genetic material from a template.

The next generation of genetic modification tools are based on sequence-specific nucleases (SSN), proteins with nuclease activity that generate sequence-specific double strand breaks (DSB).^32^ The use of SSNs in crops includes the Zinc Finger Nuclease (ZFN),^33,34^ the Transcription Activator-Like Effector Nuclease (TALEN)^35^ and the Clustered Regularly Interspaced Short Palindromic Repeats/CRISPR associated protein 9 (CRISPR/Cas9).^36^ The repair of DSBs produced by SSN can be carried out by either homology directed repair (HDR) or non-homologous end joining (NHEJ) with the latter being the more common repair mechanism (Fig. 1).^37^ Repair by NHEJ is often seamless, however on occasion errors occur leading to indels in DNA sequence that can disrupt gene function.^32^ Alternatively, the HDR repair pathway allows for the integration of an entire gene if a donor template with complementary ends to the DSB break is co-delivered with the SSN (knock-in mutant).^37^

To generate targeted mutations, it is first necessary to introduce the reagent into the cell. The most widely used methods are *Agrobacterium* and particle bombardment-mediated transformation or polyethylene glycol-mediated protoplast transfection.^21^ The reagent harboring T-DNA is randomly integrated into the genome and its components expressed by the host plant. After targeted mutagenesis is confirmed, the integrated T-DNA can be removed by self-pollination and subsequent genetic segregation. However, since self-pollination reduces heterozygosity, favors inbreeding depression, and causes loss of varietal characteristics in species with vegetative reproduction such as potato, the removal the T-DNA by genetic segregation is not ideal. Therefore, novel transformation systems that enable the delivery of a reagent without its integration into the genome such as ribonucleoprotein or nanoparticle-mediated gene delivery platforms are promising future technologies for potato gene-editing related research^38–41^

Acceptance by consumers of products derived from GMOs remains the main limitation for their widespread use. For some time, the United States Department of Agriculture (USDA) classified them as “non-organic foods,” and thus, a majority of gene edited crops have only been used for animal feed or manufacturing materials. In 2018, the USDA considered that gene editing qualified as a tool to develop non-transgenic plants and in May 2020, a new GMO regulation was issued in United States establishing several exceptions.^42,43^ Exempt from regulation are plants in which genetic modification is a deletion, a base substitution, or introduction of sequences of compatible species (*cis*-genesis).^42^ Other exceptions include when the editing method yielded identical sequences to those present in the natural gene pool or when the organism no longer carries any trace of the editing system used.^42^ These exceptions were based on the observation that particular modifications could have theoretically taken place naturally or generated through classical mutagenesis or crossbreeding. Nevertheless, the European Union maintains the previous legislation and considers edited and *cis*-genic plants as transgenic^21,42^

### Post-Transcriptional Gene Silencing by RNAi

Post-Transcriptional Gene Silencing (PTGS) or RNAi is a naturally present plant mechanism used to regulate gene expression, control developmental processes, maintain genome integrity, and defend the plant from viruses.^44–46^ RNAi is mediated by the sequence-specific degradation of target RNA transcripts that reduces its translation and therefore protein output.^44^ Taking advantage of this mechanism, a technique was developed to manipulate specific genes which could be used to modify characteristics of agronomic interest. For example, several studies have reported its potential to combat plant virus infections and insect pests.^14,44,47,48^

**Figure 2.**
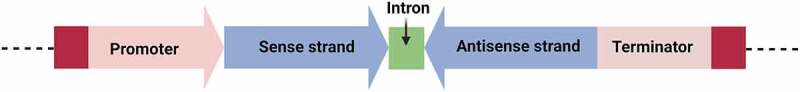
A silencing vector directed to a gene, with sense and antisense gene sequences. promoters are in sense and antisense orientation, respectively. the LB and RB elements correspond to the left and right vector borders, which is integrated into an *Agrobacterium* T-DNA. Adapted from Richael, 2021.

**Figure 3.**
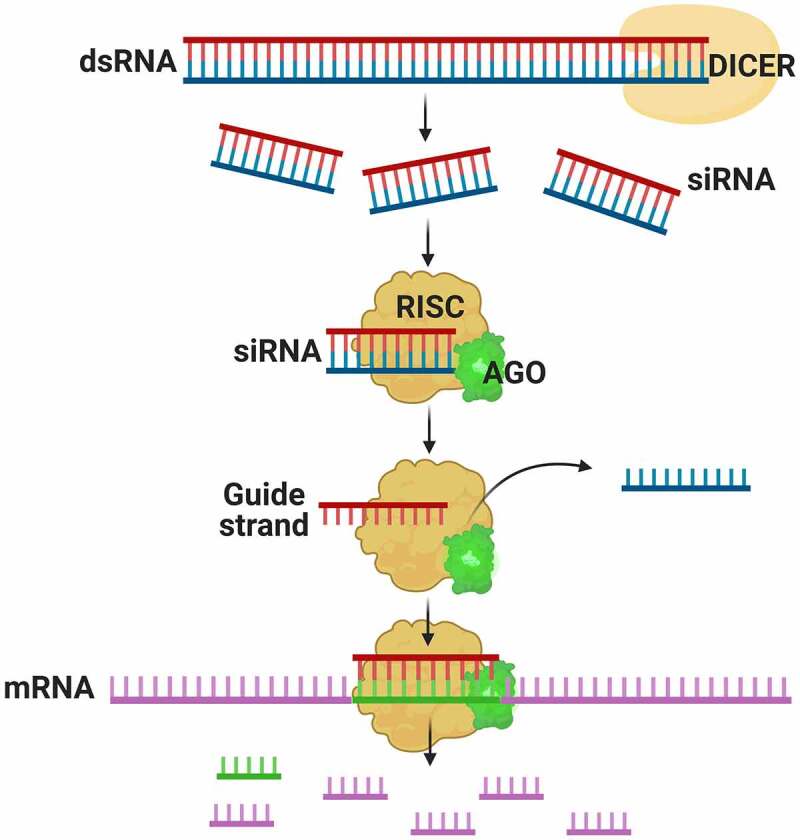
Scheme of the process of silencing a gene by interference. Introduction of a silencing vector produces a dsRNA which is processed by DICER, creating siRNAs. The siRNAs are recognized by RISC when AGO protein is joined to the complex and selects the guide strand. This strand directs the catalytic complex to the complementary mRNA, degrading it and repressing translation.

RNAi is mediated by the expression of hpRNA from an IR transgene.^31^ The IR transgene contains GOI sense and antisense sequences separated by intronic spacer sequence.^29^ When transcribed, the RNA forms a double-stranded RNA (dsRNA) molecule that is quickly degraded by the activity of DICER-Like proteins (DCL).^45^ These enzymes have RNaseIII activity that recognize dsRNA and dice it up into 21–24 nucleotide small RNA duplexes called short-interfering RNA (siRNA).^31,44,45^ Next, the siRNA duplexes are unwound and loaded into an Argonaute (AGO) enzyme, a component of the RNA-induced silencing complex (RISC) (Fig. 3). The RISC along with the target guide RNA scan the cytoplasm for complimentary RNA sequence to cleave, degrade and ultimately prevent its translation. RNAi has several advantages such as speed, efficiency, and low cost, but it does not allow complete and permanent silencing of the gene.^45^ The silencing vector can be constructed with potato genes or from sexually compatible species, resulting in a *cis*-genic modification. It also may include tissue-specific promoters, for tissue-specific silencing as opposed to constitute expression throughout the plant (Fig. 2).^44^ In addition, silencing can be enhanced by the production of secondary siRNAs that originate from a cleaved transcript converted to dsRNA by RNA-dependent RNA polymerase activity. The dsRNA is further processed by DCL to produce a cascade of secondary small RNAs that can silence multiple target transcripts.^49,50^ These siRNAs are rare in *Arabidopsis* but appear to be abundant in many crops including soybean, maize, tomato and presumably potato.^51,52^ Although it may be displaced by other techniques to achieve gene knockouts, RNAi will likely continue to be used in potato crop research and development due to its high specificity, for assessing the function of specific genes, and when down-regulation rather than complete knock out of a gene is desired.^53^

### Genome Editing with TALEN

**Figure 4.**
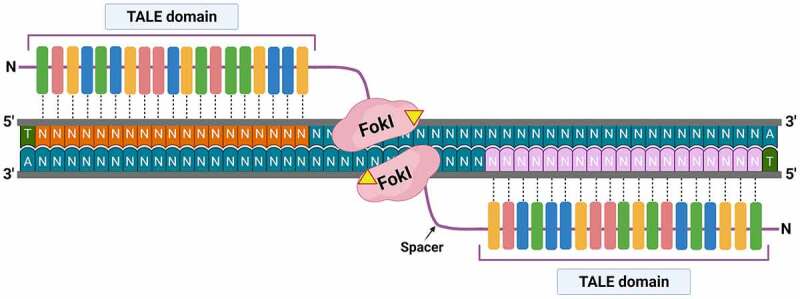
Representation of a TALEN tool attached to the DNA double helix. Yellow triangles indicate *Fok*I cut points.

TAL effectors (TALE) are DNA-binding proteins produced naturally by the bacterial pathogen *Xanthomonas* spp. When this bacteria infects the plant, TAL effectors cause the overexpression of disease susceptibility genes (*S* genes) required for disease development and bacterial multiplication.^54–56^ Based on this discovery, researchers fused the TALE proteins to the catalytic domain of the FokI endonuclease^56,57^ and laid the foundation for the gene editing tool called the TAL-effector nuclease (TALEN).^58^ Each TALEN is made up of a DNA-binding TALE domain fused to a FokI nuclease domain (Fig. 4). The TALE domain is formed by tandem repeats of 34–35 amino acid residues which recognize specific nucleotides. The di-residues at 12 and 13 of each repeat vary and are referred to as the repeat variable di-residue (RVD). The RVD binds to the major groove of the DNA double helix with high specificity while residue 12 forms a hydrogen bond that stabilizes the interaction, the residue 13 binds to a specific nucleotide. For example, the di-residue HD targets cytosine, NI targets adenine, NG targets thymine, and NN targets guanine.^57,59,60^ The catalytic domain is formed by fusion of the cleavage domain of the endonuclease FokI, which has no sequence specificity on its own.^61^ The TALEN target sequence can vary between 30 and 40 base pairs and only cleaves DNA upon dimerization of the FokI cleavage domains. This requirement gives the reagent excellent sequence specificity, and restricts off-target activity.^59^ In addition, the TALEN generates a double-stranded break that produces staggered ends which are repaired by the cellular NHEJ machinery.^26^

The TALEN reagent has many advantages including high binding efficiency and greater specificity with respect to other reagents due to the length of the recognition sequence that decreases the probability of nonspecific or off-target binding.^60^ However, the size of the editing reagent makes delivery into the cell sometimes challenging. In addition, the requirement of the TALEN pair to recognize both DNA strands makes it less convenient for multi-plexing and the simultaneous editing of multiple genes.^62^

### Genome Editing by CRISPR/Cas

**Figure 5.**
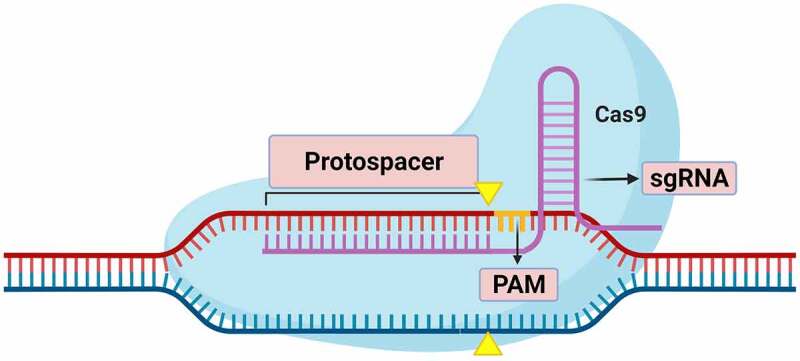
Scheme of CRISPR/Cas9 system bound to genomic DNA, forming a sgRNA-DNA heteroduplex by base pairing. Three nucleotides (Orange) represent the PAM motif, which must be recognized by the system to produce cuts. Yellow triangles mark Cas9 cut points.

The CRISPR/Cas reagent has been the most revolutionary genetic manipulation technique of the last decade. It was first discovered in bacteria, where it forms an adaptive immune system against phage viruses.^63,64^ As an editing tool it has two components: the Cas (CRISPR associated protein) nuclease, which has two lobes, REC for recognition and NUC with nuclease activity.^62,65^ It assembles with a second component, the single guide RNA (sgRNA), a non-coding single-stranded RNA complementary to the protospacer, the DNA target sequence of approximately 20 base pairs. The protospacer has at the 3ʹ end a sequence of three bases called the PAM (Protospacer Adjacent Motif) that must be recognized by the NUC domain to produce the DSB (Fig. 4). The Cas/sgRNA complex scans the double helix until it finds both a complementary sequence to the sgRNA and the PAM motif. Pairing of the complex with the DNA through the formation of sgRNA-DNA heteroduplex unwinds the DNA strands. After this, the Cas generates a DSB with blunt ends that are repaired by the cellular machinery (Fig. 5).^21,26,65,66^

The sgRNA is designed to be complementary to the target sequence for specificity and will activate once its binds with the Cas nuclease. The most widely used reagent is the Cas9 from *Streptococcus pyogenes* (*Sp*Cas9). Other Cas9 orthologs have been identified from *Staphylococcus aureus* (*Sa*Cas9),^67^ *Staphylococcus thermophillus* (*St*Cas9),^68^ and *Neisseria meningitidis* (*Nm*Cas9).^69^ Each Cas protein requires a different PAM sequence; thus, the discovery of new Cas often increases the recognition requirements. The reason for the popularity of Cas9 is due to its recognition of the 5ʹ-NGG-3ʹ sequence (N being any nucleotide, and G being guanine), a motif that occurs regularly in a given genome, allowing the editing of almost any gene.^23,62^

In polyploids crops such as potato there are several copies of each gene, requiring editing of all haplo-alleles. This increases complexity of gene editing because it is necessary to design sgRNA based on a conserved sequence among the four copies. If a conserved sequence is not found, a different sgRNA will need to be designed to target each haplo-allele.^65^ Despite this, CRISPR/Cas9 is broadly used for its simplicity and versatility compared to other reagents.^21^ Specificity can be improved, and off-target effects minimized by selecting appropriate targets using genome resources such as the recently improved doubled monoploid *S. tuberosum* Group Phureja assembly,^70^ and publicly available off-target analysis tools including the versatile Cas-OFFinder algorithm.^71^ In addition, the Cas9 and sgRNA can be assembled *in vitro*, producing a ribonucleoprotein complex, and introduced into plant cells by co-culturing protoplasted cells.^39,72^ The complex can produce an edit immediately after entering the cell and is quickly degraded by cellular proteases. It has greater editing specificity, because the complex does not require transcription and translation, reducing unwanted mutations and preventing recombinant DNA integration. This alternative is a promising tool for potato gene editing, since vegetative reproduction makes it difficult to eliminate the integrated T-DNA by segregation.^39,72^ The CRISPR/Cas9 system has been recently expanded with two new variants that allow editing of nucleotide bases without generating a DSB. These reagents are referred to as base editors and prime editors and will be described below.

## Genetically Modified Characteristics in Potato

Genetic modification has been highly successful in potato with commercialization of improved varieties for quantitative traits, such as productivity, and qualitative traits, such as disease resistance and nutritional quality.^73^ The strategies utilized include both *trans*-genesis and *cis*-genesis approaches, using either RNAi, or gene editing by CRISPR/Cas9 and TALEN technologies. A summary with the main genetically modified varieties can be found in Table 2.

**Table 2.** Summary of the most outstanding genetically modified potato varieties that have been commercialized. The data shown are developer company, commercial trade name, acquired characteristic and genetic modification, along with the first date of approval for human consumption and the country where it was approved. Table prepared by the authors with information obtained from ISAAA, 2021. Abbreviations: CAN (Canada); CIS (Cold Induced Sweetening); US (United States); EU (European Union); RU (Russia)

Developer	Commercial trade name	Accquired characteristic	Genetic modification	Approval for human consumption
Monsanto®	NewLeaf™	Colorado potato beetle resistance	*Cry3A* gene introduction	1995 (US, CAN)
	NewLeaf™ Plus	Colorado potato beetle and potato leaf roll virus resistance	*Cry3A* and PLRV replicase and helicase genes introduction	1998 (US)
	NewLeaf™ Y	Colorado potato beetle and potato virus Y resistance	*Cry3A* and PVY coat protein introduction	1998 (US)
J.R. Simplot ®	Innate® 1.0	Reduced acrylamide formation and black spot bruise	*Asn1* and *Ppo2* down-regulation	2015 (US)
	Innate® 2.0	Reduced acrylamide formation, black spot bruise and CIS; *Phytophthora infestans* resistance	*Asn1, Ppo2* and *Vlnv* down-regulation; *Rpi-vnt1* introduction	2017 (US)
BASF Plant Science	Amflora ™	Reduced amylose formation	*GBSSI* down-regulation	2010 (EU)
	Starch potato	Reduced amylose formation	*GBSSI* down-regulation	2014 (EU)
Russian Academy of Sciences	Elizaveta Plus/ Lugovskoi Plus	Colorado potato beetle resistance	*Cry3A* introduction	2005/2007 (RU)

### Starch Composition

Potato starch, composed of 80% amylopectin and 20% amylose, is synthesized in tuber amyloplasts.^74^ Amylopectin is used in various industries for the manufacture of paper and adhesives among others. However, it is necessary to remove the amylose from the starch with a chemical pre-treatment which increases costs and can causes environmental damage.^2,75,76^ Amylose is a trait with monogenic control, synthesized by the granule-bound starch synthase (GBSS). Knocked-out gene expression prevents the synthesis of amylose, producing a normal starch from the morphological point of view, but with altered chemical characteristics.^74^ This allows the direct industrial use of the amylose-free raw material.

Potato tubers producing starch with just amylopectin results in a waxy phenotype. The first amylose-free potatoes were developed by silencing the *GBSSI* gene using RNAi.^74^ More recently, transient expression of a CRISPR/Cas9 system was used to knock out the *GBSSI* gene in cv. Kuras. Mutation of the four alleles was undertaken by PEG-mediated transformation of a reagent into protoplasts, avoiding stable integration of the system. Most of the mutations observed were 1–10 bp deletions, with one base being the most recurrent.^75^ In a later work, a ribonucleoprotein complex was used for gene knock out. The regenerated plants did not express the gene, had a lower frequency of mutation outside the target, and did not have integrated exogenous DNA.^72^

Kusano et al^77^ used an improved CRISPR/Cas9 editing system with a Cas9 translation enhancer (dMac3) and multiple sgRNAs directed to *GBSSI*. The results showed that dMac3 increases translation of Cas9, which together with the introduction of multiple sgRNAs, provided increased haplo-allele mutation rates and efficiency. Transformants with the four mutated alleles were effectively obtained.

The European company BASF™ developed Amflora™ potatoes, which only contain amylopectin. Its regulation was requested for Europe in 1997 and accepted thirteen years later.^2^ However, they were only marketed for two years due to a change in the European GMO regulation.^76^ Even though amylose-free starch has high commercial value, Amflora™ potatoes were the only genetically modified potato ever marketed in Europe, approved for industrial use and animal feed.^14,77^

### Acrylamide Content

Potato tubers are kept in cold storage to increase postharvest life for up to one year. Without this procedure half-life is reduced to about six months. But low temperatures trigger an effect known as Cold Induced Sweetening (CIS) in which reducing sugars accumulate. Tubers with reducing sugars subjected to high processing temperatures form dark pigments and bitter flavors that are rejected by consumers. Reducing sugars also react with free asparagine from the tuber resulting in enzymatic browning and acrylamide production.^2,78–80^ First identified in 2002, acrylamide is formed in carbohydrate-rich foods cooked at high temperatures by the Maillard reaction between asparagine and reducing sugars, such as glucose and fructose.^81^ Studies have suggested that acrylamide is carcinogenic in rodents.^82^ Consequently, food safety agencies have established maximum values for the marketing of acrylamide-containing products for human consumption. Its implication in human health have triggered the search for alternatives to overcome this problem in potatoes. Research on acrylamide biosynthesis has identified the *Asn1, Asn2* and *Vlnv* genes as key to modifying accumulation of acrylamide.^35,83,84^

Two strategies have been pursued to limit substrates necessary for the synthesis of acrylamide in the tuber. Asparagine is the preponderant free amino acid in potatoes, representing up to 40% of the total. Its accumulation is induced by different types of stress, such as drought and salinity.^81^ Several investigations have managed to reduce asparagine concentrations by affecting the expression of the genes for asparagine synthetase 1 and 2 (*Asn1, Asn2*), on chromosomes 6 and 4, respectively. The respective enzymes catalyze asparagine formation by the transfer of the amino group from glutamine to aspartate.^35^ In one of the first studies, carried out by the company J.R. Simplot, it was suggested that lowering the expression of *Asn1* and *Asn2* was a very promising approach. An RNAi silencing vector was assembled with a conserved sequence of both genes. Expression was driven by a promoter 100 times stronger in tubers than in leaves. This vector was introduced into explants of the cv. Russet Burbank by *A. tumefaciens*-mediated transformation. Regenerated plants were cis-genic since the complex introduced consisted of gene sequences from the same species. Asparagine content was quantified, showing a concentration 20-fold lower than the control. However, transformed plants showed phenotypic changes. They produced fewer tubers that also had cracks.^84^ In another study conducted by the same company, Chawla et al.^83^ demonstrated that those developmental defects were due to the silencing of the *Asn2*. Plants that had *Asn1* as the only gene silenced showed the same reduction in asparagine concentration than those with both genes silenced, but without phenotypic differences.

These studies were key to the development of genetically modified potatoes with reduced acrylamide. The first generation Innate® potatoes were developed from cv. Russet Burbank by reducing *Asn1* expression through RNAi, using tuber-specific expression promoters. The polyphenol oxidase gene (*Ppo*) was also silenced, reducing the enzymatic browning during tuber manipulation due to release of the PPO enzyme from the plastids. As a result, the formation of asparagine, and therefore acrylamide, and the accumulation of unwanted pigments, decreased without affecting other varietal characteristics. Innate® potato was accepted for human consumption in the United States and Canada among others, in 2015 and 2016, respectively.^27,79,85^

The reducing sugars involved in acrylamide formation are glucose and fructose. In potatoes, they are produced by the hydrolysis of sucrose, mediated by the vacuolar invertase enzyme, encoded by *Vlnv* on chromosome 3. The second strategy to reduce acrylamide accumulation focuses on blocking the expression of this gene as a means to target sugars in acrylamide production.^81,86^ Initially, RNAi was used to silence the *Vlnv* in cv. Katahdin, achieving a reduction in expression of 97%.^78^ It was also confirmed that decreasing *Vlnv* expression is key to lower accumulation of the reducing sugars that trigger CIS and the synthesis of acrylamide. Nevertheless, the level of silencing achieved did not completely prevent this process.^78,81,86^ Since this approach did not allow complete silencing, and also resulted in a cis-genic plant, the company Cellectis Plant Sciences, now Calyxt Inc., used TALEN for the same purpose. This time, the expression of the gene was completely eliminated using the cv. Ranger Russet. A TALEN was designed based on the consensus sequence and the vector introduced in the plant by protoplasts transfection. Individual transformed plants having four haplo-allele knock-outs and no integrated editing cassette were selected.^35^ All mutated haplo-alleles were shown the have modifications that disrupted the reading frame resulting in loss of gene function.^35^ Chromatography analysis on regenerated plants confirmed that there was a strict correlation between the number of mutated alleles and the presence of reducing sugars in the tuber.^35,83^

This same approach for reducing acrylamide was explored by J.R. Simplot for the second generation Innate® potatoes, developed by transforming the first generation. This retransformation was carried out to decrease the expression of *Vlnv* by RNAi using the potato gene sequence, in addition to introducing a gene for resistance to *Phytophthora infestans*.^79^ These experiments found that silencing of *Asn1* is more effective than *Asn2* for reducing asparagine accumulation and that silencing *Vlnv* is more effective than Asn1 for reducing acrylamide formation. Triple gene silencing was carried out with a cassette consisting of a fragment from *Asn1* with 79% identity to *Asn2*, and another fragment from *Vlnv*. Acrylamide concentrations were similar for plants that only had *Vlnv* silenced and those that had three genes silenced. Results demonstrated that lowering the concentrations of reducing sugars affects acrylamide production more than lowering those of asparagine,^81^ establishing *Vlnv*-silencing as the best strategy to follow. *Vlnv* silencing also prevents CIS, as opposed to *Asn1* silencing. Some wild potatoes have been found not to be affected by CIS. For instance, in *S. raphanifolium, Vlnv* expression is as low as in the silenced lines, probably because either this locus has a weak promoter or due to the presence of expression suppressors. Both alternatives should be further studied to explore options to transfer these wild genes into elite varieties.^78,81^

### Resistance to Phytophthora Infestans

The oomycete *Phytophthora infestans* causes late blight disease in potato. This biotrophic pathogen invades living plant cells to obtain nutrients through the release of certain proteins, such as extracellular toxins, hydrolytic enzymes, and effectors. When the pathogen establishes on the leaf surface the effectors increase susceptibility to infection. It also produces a sporangium that generates zoospores that germinate and penetrate the tissue by haustoria.^19,87^ The first symptoms are irregular necrotic lesions in the aerial parts of the plant that spread in conducive conditions and can cause the death of the plant in days.^79^ Traditional protection against this disease is obtained by applying fungicides, but the pathogen can be found in tubers or diseased residue in the soil, thus using disease-free planting material and crop rotation is key for disease management.^88^ Late blight was responsible for the great Irish potato famine of 1845, wiping out much of the crop. Promoted by the heavy dependence on potatoes as a staple crop in Ireland, it is estimated that the disease caused a population decline of 25% between 1845 and 1849.^88,89^

Due to the significant damage caused by this pathogen, much research has been carried out to obtain resistant varieties. About 50 resistance genes (R genes) have been found against late blight through genome studies on wild potato-related species.^19^ The R proteins may confer resistance to various pathogens by recognizing intracellular effectors of avirulence (Avr) released by pathogens. This recognition triggers the plant immune response, a hypersensitive response that causes programmed death of the cells surrounding the focus of infection, preventing further disease progress. Because there are many different R genes, they provide with a wide range of protection against different pathogens. The R genes tend to share a high percentage of identity and are typically found in clusters in the genome with high rates of duplication, recombination and other genetic phenomena, which suggest a plant-pathogen parallel evolution.^89,90^ A main problem associated with R gene-mediated resistance is its durability. Resistance is lost when the pathogen evolves to evade recognition by the plant. Several strategies to lengthen resistance over time have been proposed, with gene pyramiding being the best alternative in some cases. This consists of introducing several resistance genes at the same time.^91^

However, efforts to introduce R genes into elite varieties by traditional breeding have met with little success because the approach is inherently inefficient. For instance, the potato varieties Bionica and Toluca are resistant to late blight because they carry the *Rpi-blb2* gene of *S. bulbocastanum*, but its development took more than 50 years.^19,27^ Using genetic engineering approaches, it is possible to introduce desired R genes in a more efficient and durable manner. The DuRPh (Durable Resistance against Phytophthora) program was created to identify and isolate R genes from wild related species, including their promoters and terminators, and transfer them to commercial cultivars to make them resistant through cis-genesis.^27^ Thus, in the second generation Innate® potatoes from J.R. Simplot™, the *Rpi-vnt1.1* gene from *S. venturii* was cloned into a vector driven by the native promoter and terminator. It was tested by subjecting transformed plants to the presence of the predominant *P. infestans* strains in the United States, since it would be the first region targeted for commercialization.^79^ A food safety study corroborated that the gene insertion did not cause accumulation of significant amounts of VTN1 protein in the tubers. Its presence was almost exclusively confined to the aerial part. The study also confirmed that Innate® potatoes are as safe as conventional varieties susceptible to infection, both for human consumption and for livestock.^90^

In sub-Saharan Africa *P. infestans* causes between 15–30% annual losses in potatoes. Ghislain et al.,^89^ designed a study in which three R genes were transferred from wild *Solanum* species to cultivated potatoes, achieving complete resistance. The transformed cultivars were Desirée and Victoria, and the genes chosen for transformation with *A. tumefaciens* were *RB* and *Rpi-blb2* from *S. bulbocastanum*, and *Rpi-vnt1.1* from *S. venturii*. These genes conferred broad spectrum resistance to different strains of *P. infestans*. The presence of the three R genes was shown to be more effective for plant defense than the presence of a single gene. Thus, 75% of the plants with the three R genes reached extreme resistance, compared to 3–5% of those with just one, demonstrating the validity of gene pyramiding. Plants with extreme resistance showed no symptoms and the tubers produced, used for sowing the following season, did not show symptoms of the disease. In addition, the introgression of these genes did not cause phenotypic changes or alter productivity.

Due to the fact that some strains can evade the recognition of R proteins, other approaches have focused on finding new immune receptors against late blight. For instance, *P. infestans* has been found to have six genes for elicitin, a highly conserved extracellular protein. In *S. microdontum*, the elicitin response protein (ELR) recognizes the pathogen elicitin as a pathogen-associated molecular pattern and triggers an immune response. Transforming Desirée variety with the *ELR* gene has been shown to increase resistance to a wide range of *P. infestans* strains.^92^

Using new gene editing techniques, such as base editing and prime editing, R genes could be edited by creating non-synonymous base changes in essential amino acids to recognize the pathogen more efficiently, increasing specificity.^21^ Improving resistance to late blight is extremely important and could significantly reduce the use of pesticides and the economic losses worldwide. Compared to the use of fungicides, resistance in commercial varieties is the most sustainable solution to control the disease. Research suggests that better results could be achieved if extracellular and intracellular recognition can be complemented, making the resistance generated broader and more stable.^27,89,92^

### Resistance to Colorado Potato Beetle

Colorado potato beetle is one of the major pests affecting potato crops. The defoliating insect is native to North America and has since spread to Europe and other parts of the world. Both adult and larval stages feed on potato leaves which in turn affect the tuber resulting in large yield losses.^14^ Several insecticidal treatments can be applied throughout the growing season to avoid these losses, however this often increases production costs and environmental impact, since treatments also cause the death of some beneficial insects.^14^ In addition, the insect has developed resistance to active ingredients of many of the synthetic insecticides used, including numerous pyrethrins.^93,94^ These issues have prompted a search for alternative pest management strategies.

Synthesized by the *CryIIIA* gene of *Bacillus thuringiensis*, the Bt protein causes the selective death of coleopterans of the Chrysomelidae family, including the Colorado potato beetle. In its normal state, Bt is an inactive protoxin that is activated by both serine proteases and basic pH in the insect gut. Once activated it binds to specific receptors on the intestinal epithelium and opens membrane cation channels, causing the lysis of digestive tract cells and death of the insect by starvation.^14^ The first genetically engineered Bt-potatoes were developed by Monsanto™ under the name NewLeaf™. A transformation vector was created and cloned into *A. tumefaciens*, in which the *CryIIIA* gene was driven by the cauliflower mosaic virus (CaMV) 35S promoter. Russet Burbank, Superior, and Atlantic potato varieties were each transformed with *CryIIIA* cassette, resulting in expression of the Bt protein in their leaves. In addition to causing high beetle mortality, a drastic reduction in the ovary size of female Colorado potato beetles was observed, affecting reproduction.^85,94^ All three potato varieties were accepted by the USDA for commercialization in the United States in 1995, and crop production reached 55,000 hectares by 1998. In 1998 Monsanto released NewLeaf Plus™ potatoes developed from the cv. Russet Burbank, which had added potato leafroll virus (PLRV) resistance.^93^ However, by 2001 commercialization had halted due to low profits most likely triggered by the rejection of GM potato by food companies.^2^

The advantages of NewLeaf™ potatoes included the high specificity of Bt protein for coleopterans that had no effect on predator species such as spiders and hemipterans, and the reduced use of costly synthetic and environmentally harmful insecticides. Although, beetles may develop resistance to this control method, it has been demonstrated that commercial preparations of Bt are not as effective because, of their photosensitivity and because preparations are often washed off by rain or irrigation.^14^

Glycoalkaloids are compounds often targeted for reduction in potatoes because they can add bitterness and are toxic to humans. Predominant glycoalkaloids in current potato cultivars are α-solanine and α-chaconine, which account for 90% of the total.^95–97^ However, they also act as deterrents, minimizing the attack of herbivorous insects such as the Colorado potato beetle. Thus, their reduction in the aerial part of the plant may have led to an increased susceptibility to pests. Because accumulation of glycoalkaloids varies within the plant, there is an interest in silencing only the biosynthetic pathway in the tuber, and in increasing levels of glycoalkaloids in the foliage. Leptins and leptinins are glycoalkaloids found in *S. chacoense*, which only accumulate in aerial organs. These glycoalkaloids were found to be positively correlated with resistance to Colorado potato beetle,^98^ and are potentially a source of genetic resistance that merits further exploration.

A novel strategy for Colorado potato beetle resistance involves^99^ modified potatoes using RNAi technology that expresses dsRNA in chloroplasts targeting the beetle β-actin gene.^99^ Chloroplasts lack RNA silencing machinery and cannot break down dsRNA, causing its accumulation. When the insect feeds on the leaves, the dsRNA is released from the chloroplasts and is taken up by the cells of the intestine, where it degrades the β-actin mRNA by post-transcriptional gene silencing (PTGS). This approach generated a high beetle mortality with reduced foliar biomass loss and offered protection without expressing the dsRNA in the tubers. However, as insects do not have RNA-dependent RNA polymerase genes, the dsRNAs are not amplified, so the affected insect cells are only those that take up the ingested dsRNA. Thus, the plant would have to produce and store a large amount of dsRNA for efficient beetle management. Development of similar strategies based on PTGS of essential genes for the insect is expected to revolutionize pest control.

### Resistance to Herbicides

Herbicides are chemical compounds used in agriculture to eliminate weeds that compete with crops. In cultivated potato, losses due to weeds are estimated between 16% and 76%.^100^ Obtaining crops resistant to herbicides is important because some herbicides may also affect productivity of the crop. Herbicide resistant cultivars make it possible to manage competing weeds without affecting the cultivated plant. Studies on genes involved in the action of herbicides in potato have been carried out mainly to assess transformation efficiencies or to develop editing strategies. For instance, Butler *et al*.^101,102^ added mutations in the acetolactate synthase 1 (*ALS1*) gene using combinations of either CRISPR/Cas9 or TALEN and a donor template provided by a geminiviral replicon (GVR). The main objective for these studies was to demonstrate that using a GVR to deliver a donor template for HDR repair is efficient in potato and that the mutations generated are heritable. Another example targeting the same gene proved that a transiently expressed TALEN system is a valid approach to edit tetraploid cultures.^103^ *ALS1* synthesizes the acetolactate synthase enzyme, which is part of pathway that makes valine, leucine, and isoleucine in the plant. This metabolic route is the target of strong herbicides such as sulfunylureas and imidazolinones, because blocking the synthesis of those amino acids greatly reduces the synthesis of proteins, causing serious deficiencies in plants.

Other studies have focused on incorporating resistance to glyphosate. Glyphosate is the active principle of the most widely used herbicides worldwide, so development of glyphosate-resistant plants could have a great economic impact. Its mechanism of action is based on inhibiting the action of EPSP synthase, an enzyme that is only present in bacteria, fungi, and plants, preventing the formation of aromatic amino acids: phenylalanine, tryptophan, and tyrosine. Glyphosate displaces the natural enzymatic substrate, phosphoenolpyruvate (PEP), preventing the formation of the EPSP intermediate. Bakhsh et al.^100^ modified four potato cultivars for resistance to glyphosate by integrating the bacterial *CP4-EPSPS*. The enzyme C4-EPSP, isolated from *Agrobacterium*, is insensitive to the herbicide due to a change in conformational structure.^104^

Since the onset of bioengineering, there have been numerous efforts to develop herbicide resistant crops, with glyphosate resistant soybeans the first to be commercialized in 1996. Other herbicide resistant crops such as glyphosate resistant cotton and corn have also been grown widely. However, herbicide resistance has not been commercialized in potato,^100^ but the trait has great potential to reduce production costs and improve environmental sustainability of the crop.

## Future Perspectives in Potato Gene Editing

The continued optimization of gene editing platforms will increase their potency and effectiveness and facilitate more advanced gene edits. This will be necessary to address the challenges of gene editing in crops with higher ploidy levels, especially auto-tetraploids. For example, screening plants for multiple haplo-allele mutations in multiple genes can be notoriously challenging.^105,106^ The availability of more affordable long read sequencing technologies such as PacBio® amplicon sequencing and Nanopore® will greatly help mutant characterization. In addition, utilizing technologies that limit transgene integration without reducing reagent efficiency will be an important technical goal. Current efforts include the delivery ribonucleoproteins to generate transgene-free edits but should be expanded to include other promising technologies such as nanoparticle-mediated gene delivery^40,41^ or negative selectable markers such as the bacterial *coda* gene.^107^

**Figure 6.**
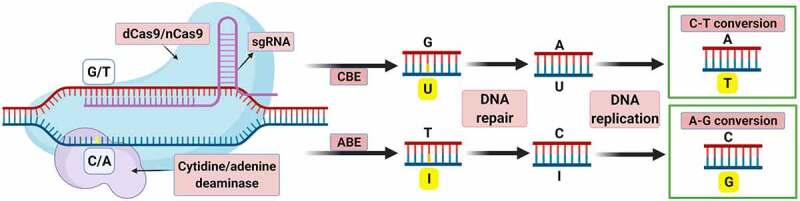
Graphic representation of the mode of action of cytosine (CBE) and adenine (ABE) base editors. The editor component is attached to complementary genomic DNA, producing directed deamination of either cytosine or adenine, respectively. Subsequently, the edited strand is permanently repaired after DNA replication, fixing the base change. Modified from Mishra et al.^108^

Several advancements have seen modifications to the CRISPR/Cas9 reagent that has led to the development of novel gene editing tools called base editors (BE) and prime editors (PE). These reagents do not require DSB or utilize the DNA repair mechanism to generate the modifications.^37^ The BE allows replacement of a single nucleotide base without requirement for a DSB or DNA donor template. This is achieved by modifying the Cas9 protein in two different ways. First, suppressing its nuclease activity (dead Cas9, dCas9) and secondly by fusing a cytidine deaminase to the editing reagent. Deamination of cytidine converts the single-stranded target C to U. The resulting G:U heteroduplex can be permanently converted to an A:T base pair after DNA replication or repair. The complex has a fused cytidine deaminase in the cytosine editors (CBE) or an adenine deaminase in the adenine editors (ABE). Both lead to base transitions: a substitution of thymine for cytosine in CBEs, while adenine for guanine in ABEs (Fig. 6). Base editing has been shown to produce less off-target effects and to be highly efficient, though bystander mutations are commonplace and problematic.^37^ It is very useful for inducing point mutations, which can help modify some important agronomic characteristics. However, these methods cannot be used to replace or insert gene sequences, have significant target restrictions and cannot convert all nucleotide bases.^37,108,109^ However, in tetraploid potato four copies of the *GBSSI* gene have been edited with this technique to eliminate amylose synthesis, demonstrating the utility of this tool.^76^ A CBE was used to induce a base mutation, which caused a loss of function of the alleles. The system was introduced by transfection of protoplasts with transient expression, resulting in non-transgenic regenerated plants.

**Figure 7.**
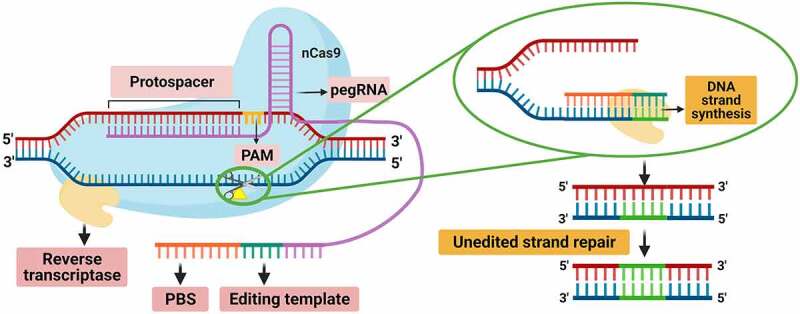
Schematic representation of the prime editing tool. The editing system is attached to complementary target DNA by pegRNA, after which nCas9 produces a nick in the 3ʹ-5ʹ strand. Subsequently, reverse transcriptase uses the cut strand as a primer to synthesize the new edited strand from the pegRNA template. The edited strand hybridizes with the unedited one, which is preferentially repaired based on the edit. Modified from Marzec and Hensel.^111^

Recently, Anzalone et al.^110^ developed a technique called prime editing (Fig. 7), which allows for the introduction of targeted and precise insertions, deletions, and all twelve types of point mutations without requiring a DSB or DNA donor template. Lin et al.^112^ successfully applied prime editing in rice and wheat, and promising results have been demonstrated in maize.^113^ In this technique, the Cas9 has nickase activity (nCas9), which cuts only the strand to edit, and the sgRNA is replaced by pegRNA (prime editing guide RNA), which directs the nCas9 to the target, and also contains the sequence to be edited and a primer binding site (PBS).^111,112^ The nCas9 is fused with a reverse transcriptase (RT) that utilizes pegRNA as a template to synthesize a complementary DNA strand. The new strand is copied directly from the pegRNA, after which the DNA is repaired, incorporating the new sequence permanently.^112^ Prime editing can do all possible transversions, and the specificity is much higher than with any other tool because DNA hybridizes with pegRNA, PBS, and the RT product, achieving fewer off-target mutations.^111^ These recently developed tools, especially prime editing, will require more optimization to understand the optimal pegRNA design rules and to overcome inconsistent efficiencies.^37^

## Conclusions

Commercial success of genetically modified potatoes must prioritize traits that benefit producers and consumers and, at the same time, reduce the environmental impact of production. For example, amylose-free potato varieties, such as Amflora™, benefit industry and help preserve the environment because they produce starch that does not require chemical pretreatment. Research is needed to determine the most effective gene targets for each trait. For instance, exploring several target genes for lowering acrylamide content identified knock outs of *Vlnv* as the most effective strategy. However, as several studies have pointed out, the best strategy to control Colorado potato beetle pests is unclear. Reducing insecticides and fungicides will increase agricultural sustainability. Future efforts should concentrate on developing potatoes with durable resistance to pests and diseases. For instance, increasing glycoalkaloids in the foliage appears to be effective against Colorado potato beetle, while protection from *P. infestans* will perhaps require pyramiding or editing of R genes.

We anticipate that the CRISPR gene editing system will continue to be the most widely used technique, since it allows for a broad range of edits to be made with great precision. In potato, base and prime editing, have great potential. However, as novel tools, specific protocols should be developed if needed. Tools that do not leave traces of exogenous DNA should be prioritized, since they have fewer regulatory hurdles. In this regard, an investigation of the traits modified by RNAi using gene editing is warranted. Additionally, screening germplasm of related wild species will continue to be crucial to identify traits in natural gene pools ready for introgression in potato cultivars.

Finally, development of improved varieties must strike a balance between benefits and public acceptance. For this, broad and clear scientific dissemination is necessary on both GMOs and techniques used, focusing on the scientific evidence of their safety. GMOs have been repeatedly rejected in some countries, with a pronounced decline in acceptance during the last decade. The deregulation of gene edited plants in the United States is a positive step toward the wider availability of crops that have improved agricultural sustainability and could benefit the hungry around the world.

## Disclaimer

Mention of any trade name, proprietary product or specific equipment does not constitute a guarantee or warranty by USDA-ARS and does not imply its approval to the exclusion of other products that may also be suitable. The USDA-ARS is an equal opportunity and affirmative action employer and all agency services are available without discrimination
